# Lymphoepithelioma-Like Carcinoma of the Breast: A Case Report of a Rare Type of Invasive Carcinoma

**DOI:** 10.7759/cureus.29231

**Published:** 2022-09-16

**Authors:** Sara Salehiazar, Hehua Huang, Maryam Aghighi, Rose Venegas

**Affiliations:** 1 Pathology and Laboratory Medicine, Harbor-UCLA (University of California Los Angeles) Medical Center, Torrance, USA; 2 Pathology, Harbor-UCLA (University of California Los Angeles) Medical Center, Torrance, USA

**Keywords:** breast invasive carcinoma, triple-negative breast carcinoma, good prognosis, medullary lymphoma carcinoma, lymphoepithelioma-like carcinoma

## Abstract

Lymphoepithelioma carcinoma (LELC) is an extremely rare type of mammary cancer. Based on the histology, it can be misdiagnosed with inflammatory lesions like mastitis and medullary carcinoma or other hematopoietic neoplasms like lymphoma in the breast. Since LELC has a good response to chemotherapy with a good prognosis, t is prognostically important to recognize LELC.

We report a rare case of LELC in a 51-year-old pre-menopausal female with a left breast mass, diagnosed with invasive ductal carcinoma (IDC), LELC type, treated with mastectomy, followed by adjuvant chemotherapy and radiotherapy, with a disease-free interval of 10 months. Herein, we present the case with its clinical presentation, radiologic imaging, histopathological features, and immunohistochemistry (IHC) findings. The rarity of this type of breast tumor warrants studying the behavior of these uncommon tumors to avoid misdiagnosis and establish well-defined criteria for diagnosis.

## Introduction

Lymphoepithelioma-like carcinoma (LELC) is an undifferentiated carcinoma with lymphocytic infiltration between tumor cells. LELC was first described by Regaud and Schminke in the nasopharynx in 1921 [1]. However, the first breast carcinoma case with the histopathologic features of LELC was first defined by Kumar and Kumar in 1994 [2,3]. Although most LELC types of cancers are related to the Epstein-Barr virus (EBV) positivity, none of the published breast cases was EBV positive [4] and mostly had a good prognosis [5]. On histology, the tumor cells appear as sheets of malignant epithelial cells with eosinophilic cytoplasm in the background of an inflammatory infiltrate. The inflammatory cells consist of lymphocytes and plasma cells, which are sometimes separated by fat and walled by hyaline septate hyphae [6]. Since LELC of the breast is an uncommon tumor type, the diagnosis may be challenging due to the similarity of histologic features with medullary carcinoma and invasive ductal or lobular lymphocytic invasive carcinoma.

Here, we report a case of LELC that invaded the pectoralis muscle with histopathologic features and IHC features that can be helpful to get the correct diagnosis since the LELC type has a better prognosis [5]. To our knowledge, from 1994 until now, approximately 35 cases have been reported [7,8] but none of them presented with invasive LELC to the adjacent muscle.

## Case presentation

**Figure 1:**
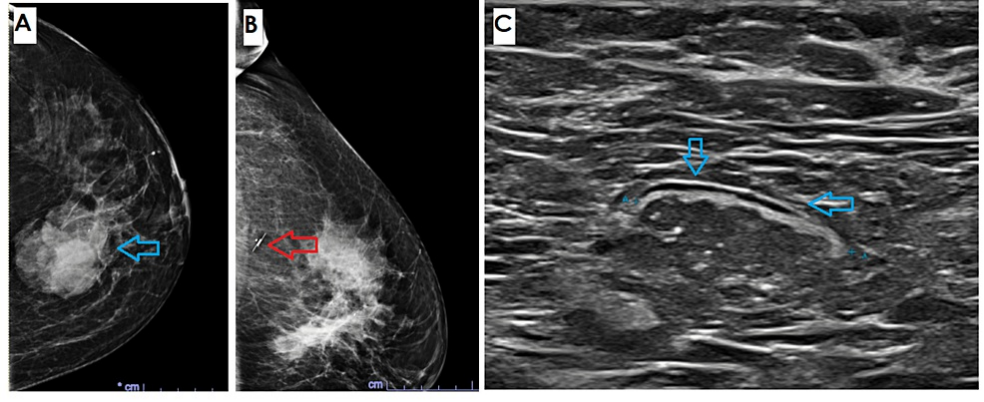
Image A: Mammographic images demonstrate a 6.4 cm, irregular-shaped mass (blue arrow). The mass is hyperdense with an irregular margin; no calcifications are present. The mass was categorized as BI-RADS category 5: highly suggestive of malignancy. Image B: Localized clip from the biopsy (red arrow). Image C: A solid oval mass measuring 2.5 cm in the pectoralis muscle BI-RADS: Breast Imaging-Reporting and Data System

The patient was a 51-year-old Hispanic female with a left breast mass, who complained of breast fullness and pain in the left axilla. Her past medical history showed obesity (BMI 30+), hypothyroidism, and vitamin D deficiency. Imaging revealed an irregular vertical mass with angular margins, measuring 6.4 x 5.8 x 4.1 cm in the left breast at 11 o'clock, located 9 centimeters from the nipple (Figures 1A-1B), and a solid oval 2.5 cm mass in the pectoralis muscle (Figure 1C). The masses were categorized as Breast Imaging-Reporting and Data System (BI-RADS)-5.

**Figure 2:**
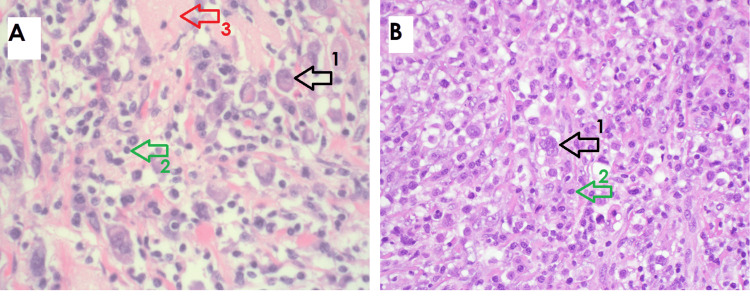
Image A: Breast mass; sheets of tumor cells (black arrow), mixed with lymphocytic stromal infiltrate (green arrow). Scattered hyaline material (red arrow) was noted;(H&E, magnification ×400). Image B: Pectoralis mass; the tumor cells (black arrow) showed similar histology as the breast mass; sheets of neoplastic cells intermingled with stromal lymphoid cell infiltrates (green arrow) (H&E, magnification ×200).

**Figure 3:**
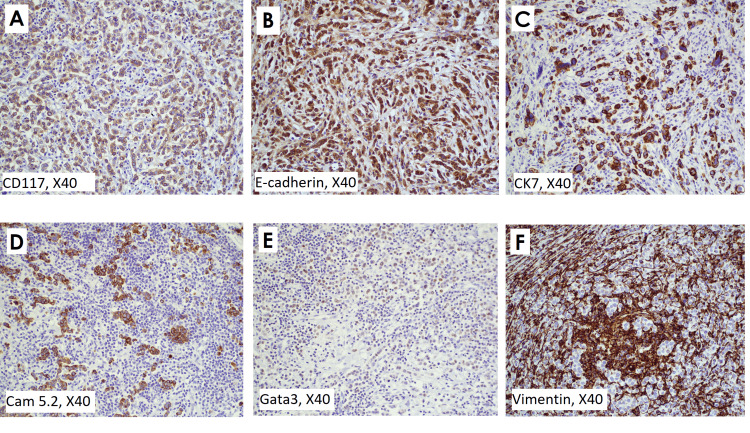
Tumor cells are strongly positive for CD117 (C-kit) (A), E-cadherin(B), CK7(C), and Cam 5.2(D), which support the epithelioid nature of the tumor cells. Mammary-type tumor cells are focally positive for Gata3 (E). Vimentin (F) is negative in tumor cells and strongly positive in stroma and lymphocytes.

The patient underwent a core biopsy of this lesion and was diagnosed as a poorly differentiated infiltrating carcinoma with a Scarff-Bloom-Richardson (SBR) score of 8 (Grade III), negative for HER2 amplification by IHC and fluorescence-in-situ hybridization (FISH) at an outside institution in early 2021, prior to presentation at our institution. She underwent a left breast simple mastectomy at our institution in late 2021, which grossly revealed a 5.5 x 4.5 x 1.5 cm white mass in the upper inner quadrant containing a localization clip from the prior biopsy. Also, submitted separately was an intra-pectoral muscle mass measuring 2.8 x 2.5 x 2.5 cm because of concern for invading the tumor to the pectoralis muscle along with seven sentinel lymph nodes. Sentinel nodes were all negative for carcinoma by hematoxylin-eosin (H&E) and Cam 5.2 IHC. The histology of both breast and pectoral mass showed tumor cells settled in a trabecular pattern with a prominent lymphocytic infiltrate intermingle with epithelial cells (Figures 2A-2B). The malignant epithelial cells were round to oval with granular ample cytoplasm and a large nucleus. No lymphovascular invasion or intraductal component was noted. The tumor was histologic grade III. The IHC stains study showed the expression of the following antibodies: E-cadherin, cytokeratin 7 (CK7), and cluster of differentiation 117 (CD117) (Figures 3A-3C) in tumor cells. Cam 5.2 was patchy positive, Gata-3 focal weakly positive in epithelioid tumor cells (Figures 3D-3E), estrogen-receptor (ER) and progesterone-receptor (PR) negative, and Vimentin only positive in the stroma and lymphocytes (Figure 3F).

As a result of the histopathological and IHCs findings, this case was diagnosed as ductal carcinoma lymphoepithelioma-like, and the tumor size in the greatest dimension was 20 mm with tumor invasion to the pectoralis muscle in the absence of invasion of the chest wall structure and was so staged as mypT4apN0. Breast biomarkers ER (0%), PR (0%), and HER2 by in situ hybridization were negative. Following surgery, the patient completed eight courses of chemotherapy followed by radiotherapy. At 10 months postoperative, the patient was tumor-free, with no evidence of recurrent disease.

## Discussion

Lymphoepithelioma-like carcinoma (LELC) is an uncommon tumor type consistent with undifferentiated carcinoma with a melded reactive lymphocytic infiltrate. In other words, LELC carcinomas arise from tissues other than the nasopharynx but have morphologic characteristics similar to those of nasopharyngeal lymphoepithelioma [8]. Although EBV has been linked to the pathogenesis of LELC of the nasopharynx, salivary glands, stomach, and others, it has never been associated with breast LELC [1,4]. As a result, the etiology of the LELC is not clear to be related to viral infections, however, there is a slight possibility of related LELC to human papillomavirus (HPV). To our knowledge, HPV has been detected in only three cases of IDC with LELC type [4,5]. Our case did not check for HPV and EBV due to the unavailability of antibodies.

The ER, PR, and Her2 receptors are negative in most cases so LELC can be considered part of the triple-negative breast cancers (TNBC) in consideration of positive cytokeratin 5/6 in basal cells [9]. Remarkably, LELC can express CD117 as a cell-surface marker. CD117 is a proto-oncogene and is a receptor for cytokine stem cell factors, so cellular signaling through CD117 has an important role in cell survival, proliferation, and differentiation. However, the significance of CD117 expression in LELC of the breast, with its prognostic and therapeutic implications, needs more study [8].

CD 117 is a helpful IHC to highlight the LELC-type tumor cells though the expression of CD117 is more frequent in medullary cancers [10]. Nodal involvement and distant metastasis are extremely rare and LELC has a good response to chemotherapy with a good prognosis [9], and this case is the only LELC type of breast cancer with tumor invasion to the pectoralis muscle, which responded very well to chemotherapy, mastectomy, mass excision, and radiotherapy. Therefore, it is prognostically important to recognize LELC. The differential diagnosis of LELC includes medullary carcinoma and particular types of lymphoma and, rarely, metastasis from the nasopharynx [3,9]. It can be challenging to distinguish LELC from medullary carcinoma or a specific type of lymphoma like Hodgkin lymphoma. However, histologically, Hodgkin lymphoma consists of small to medium-sized lymphocytes with scant basophilic cytoplasm mixed with eosinophils, plasma cells, and scattered large pleomorphic Reed Stenberg or anaplastic giant cells (Hodgkin cells). Medullary carcinomas are well-circumscribed and present in adjacent breast tissue rather than invade it, but LELC is an undifferentiated carcinoma in the background of reactive lymphocytic infiltrate. Also, a decreased plasma cell component infiltrate in stroma favors the LELC diagnosis. Additionally, IHC stains are accommodating the diagnosis of LELC. Cytokeratin (CKs) and EMA can easily highlight the epithelium component of tumor cells, which can be obscured by lymphoid components. The lymphoid component in most cases of LELC has T lymphocytes (CD3+, CD8+) mixed with some B lymphocytes. In addition, medullary carcinoma has a syncytial growth pattern of more than 75%, complete circumscription, diffuse mononuclear stromal infiltrate, moderate to marked nuclear pleomorphism, and absence of microglandular features [11,12].

## Conclusions

In conclusion, LELC of the breast is a rare tumor with specific morphological features and mostly has a good prognosis. Its pathogenesis remains unclear and likely multifactorial. There is some evidence regarding the possible association between EBV and HPV; however, most LELC is negative for EBV-encoded small nuclear RNA and only a few of the LELC cases are positive for HPV. To the best of our perception, this is the thirty-fifth case report of this entity, and the majority of reported LELC cases are pre-menopausal or menopausal and TNBC with rare metastasis. The correct diagnosis of LELC can be achieved with a careful evaluation of the histopathology and link with IHC findings. Since LELC is an uncommon diagnosis of breast invasive carcinoma and probably will be reported more; consequently, certain guidelines and diagnostic criteria for the diagnosis of LELC in the breast are reasonable.
